# Preparation of novel polyamine-type chelating resin with hyperbranched structures and its adsorption performance

**DOI:** 10.1098/rsos.171665

**Published:** 2018-02-07

**Authors:** Youning Chen, Wei Zhao, Huan Wang, Yuhong Li, Chenxi Li

**Affiliations:** College of Chemistry and Chemical Engineering, Xianyang Normal College, Xianyang 712000, People's Republic of China

**Keywords:** surface-initiated atom transfer radical polymerization, polyamine-type chelating resin, heavy metal ions, adsorption

## Abstract

This paper explored the method of combining atom transfer radical polymerization (ATRP) technology and hyperbranched polymer principle to prepare the high capacity chelating resin. First, surface-initiated atom transfer radical polymerization (SI-ATRP) method was used to graft glycidyl methacrylate (GMA) on chloromethylated cross-linked styrene-divinylbenzene resin, and then the novel polyamine chelating resin with a kind of hyperbranched structure was prepared through the amination reaction between amino group of (2-aminoethyl) triamine and epoxy group in GMA. This resin had a selective effect on As(V) and Cr(VI) at a relatively low pH and can be used for the disposal of waste water containing As(V) and Cr(VI). It had a relatively strong adsorption effect on Cu(II), Pb(II), Cd(II) and Cr(III) and can be used for the disposal of heavy metal ion waste water. The finding was that, the adsorption capacity of resin on the studied heavy metal ions was higher than that of the chelating resin synthesized by traditional technology and also higher than that of the resin modified by ATRP technology and bifunctional chelator, indicating that the combination of ATRP and hyperbranched polymer concept is an effective method to prepare chelating resin with high capacity.

## 1. Introduction

As amino has strong chelating performance on transition metal ions, the adsorbent with amino as the functional group is widely applicable to the removal of heavy metal ions in solution [1–7]. By means of the theory of hard and soft acid and base (HSAB), Lam *et al*. [8] studied the adsorption selectivity of MCM-41 adsorbent (NH_2_-MCM-41) on Ag^+^ and Cu^2+^; by comparing the distribution coefficients (*K*_d_) of heavy ions in amino-functional activated carbon (NH_2_-AC), Yantasee *et al*. [9] investigated the adsorption selectivity of NH_2_-AC on four kinds of heavy metal ions. These studies mainly focused on the selectivity, and the study on how to improve the amino density was rarely reported. With the emergence of atom transfer radical polymerization (ATRP) technology, Deng *et al*. [10] adopted surface-initiated atom transfer radical polymerization (SI-ATRP) and managed to graft glycidyl methacrylate (GMA) onto the PS surface, then used ethylidene diamine for amination, and finally obtained aminated resin. The adsorption capacity of this resin on Cu(II), Pb(II), Cr(VI) and As(V) was 2.6 mmol g^−1^, 0.97 mmol g^−1^, 3.0 mmol g^−1^and 2.2 mmol g^−1^, respectively. Although its adsorption capacity is higher than that of the traditional resin, the investigators of this study applied one amino in the ethylene diamine to the ring opening reaction and only one remaining amino was involved in the chelation of metal ions, so it was difficult to improve the number and adsorption capacity of the functional groups grafted on the material surface; in order to improve the adsorption capacity, new approaches should be adopted.

During recent years, the synthesis of hyperbranched polymers has been very studied vigorously [11,12]. Hyperbranched polymer is a type of highly irregular multi-level branched polymers with three-dimensional spherical structures and rich terminal groups attached at the end of the polymer [13–15]. This terminal group can serve as a functional group or chelation group for further reaction to provide a possibility for the increase of active groups on the polymer surface [16,17]. This paper combines ATRP technology and the concept of hyperbranched polymer and proposes a new method to prepare a kind of high capacity chelating resin. First, SI-ATRP was used to graft the GMA onto the surface of CMPS resin, then the cycle addition reaction was performed through the amino in tris(aminoethyl)amine and the epoxy group in GMA to prepare the polyamine chelating resin with hyperbranched structures, and its adsorption performance on the As(V), Cr(VI), Cu(II), Pb(II), Cd(II) and Cr(III) in solution was investigated.

## 2. Experiment

### 2.1. Reagents and devices

Chloromethylated polystyrene (Xi'an Lanxiao Science and Technology New Material Co., Ltd, with a chlorinity of 18% (mass fraction), a content of cross-linking agents of 6% (mass fraction)); GMA (CP, Shanghai Aladdin); cuprous bromide (CP, Tianjin Chemical Reagent Factory); 2,2′-bipyridine (CP, Tianjin Chemical Reagent Factory); tetrahydrofuran (THF) (CP, Tianjin Hedong District Hongyan Reagent); tris(aminoethyl)amine (AR, Shanghai Aladdin); acetone (CP, Luoyang Chemical Reagent Factory); methanol (CP, Tianjin Hedong District Hongyan Reagent); ethanol (CP, Luoyang Chemical Reagent Factory); Na_2_HAsO_4_·7H_2_O (AR, Tianjin Fuchen Chemical Reagent Factory); K_2_Cr_2_O_7_ (AR, Tianjin Fuchen Chemical Reagent Factory); Cr(NO_3_)_3_·9H_2_O (AR, Tianjin Fuchen Chemical Reagent Factory); Cd(NO_3_)_2_·4H_2_O (AR, Tianjin Fuchen Chemical Reagent Factory).

TENSOR type 27 Fourier transform infrared spectrometer (Bruker, Germany); Rario type II elemental analyser (Elementar, Germany); novAA400 atomic absorption spectrometer (Analytik Jena AG, Germany); KYKY-2800B scanning electron microscope (KYKY Technology Co., Ltd, Beijing); K-Alpha X-ray electron spectrometer (Termo Fisher, USA); Micromeritics ASAP 2010 (Georgia USA).

### 2.2. Surface-initiated atom transfer radical polymerization of glycidyl methacrylate

(1) Five grams of chloromethylated polystyrene (macromolecular initiator), 0.5 g of cuprous bromide, 50 ml of THF and 10 ml of GMA; after freezing and evacuation, nitrogen gas was put in for three cycles, and reacted at 40°C for a period of time.
(2) Removal of copper ion: the products were washed using acetone, then they were stirred in 10% EDTA (EDTA/ethanol volume ratio: 1 : 1) for reaction for 24 h, then they were filtered and washed with water, and finally with ethanol. They were dried at vacuum and 35°C conditions.

The polymerization time and monomer dosage were used to control the degree of grafting (DG) of GMA in CMPS. DG calculation method is as follows:

2.1 $$\text{DG}(\%)=\frac{W_{a}-W_{b}}{W_{b}}\times 100\%,$$

*W*_a_, mass of resin after reaction (g); *W*_b_, mass of resin before reaction (g).

### 2.3. Synthesis of polyamine chelating resin

Five grams of GMA grafted resin and 50 ml of THF were added to a 250 ml three-neck flask equipped with a motor stirrer, thermometer and reflux condensing tube, and then stirred evenly, 15 ml of tri(2-aminoethyl)amine was added, then the solution was heated in a water bath to 50°C, and it was stirred to allow reflux and reaction for 8 h. After the reaction ended, the reactants were cooled to room temperature, the reaction solution was filtered, then ethanol was used for drip washing, then it was washed with deionized water to such an extent that the phenolphthalein indicator did not become discoloured through the efflux, and finally it was vacuum dried at 35°C to obtain the polyamine chelating resin.

### 2.4. Structural characterization of resin

Tensor 27 FT-IR infrared spectrum (Bruker Company, Germany) and X-ray photoelectron spectroscopy (XPS, PE, PHI-5400, USA) for the characterization of CMPS, PGMA-g-PS and multi-aminated resin.

### 2.5. Batch adsorption test

The adsorption performance of resin on As(V), Cr(VI), Cu(II), Pb(II), Cd(II) and Cr(III) was studied; under the conditions of no competition, the resin was placed into the solution containing one kind of metal ion for adsorption.

#### 2.5.1. Effect of pH

A series of 100 ml solutions with different pH values at an initial concentration of 7 mmol l^−1^ containing As(V), Cr(VI), Cu(II), Pb(II), Cd(II) and Cr(III) were added to 0.1 g of resin. The range of change of the pH value of As(V) and Cr(VI) was 1.0–12.0; the range of change of the pH value of Cu(II), Pb(II), Cd(II) and Cr(III) was 1.0–7.0. The mixture was placed onto a constant temperature oscillator, and constant temperature oscillation was performed at the rate of 200 r.p.m. at the designated temperature. After the adsorption was completed, 0.2 µm membrane filtration was performed to separate the resin, the atomic absorption spectrum (AAS) method was used to determine the concentration of the metal ions in filtrate, and the adsorptive capacity is calculated as follows:

2.2 $$Q=\frac{(C_{0}-C)V}{W},$$

where *Q* is the adsorptive capacity (mmol g^−1^); *C*_0_ and *C* represented the initial concentration of metal ions in solution and the concentration of those after adsorption (mmol l^−1^); *V* is the volume (litre); *W* is the resin mass (gram)

#### 2.5.2. Adsorption isotherm

The pH value of the stock solution of Cu(II), Pb(II), Cd(II) and Cr(III) was adjusted to 5.0, the pH value of the stock solution of As(V) was adjusted to 4.0, the pH value of the stock solution of Cr(VI) was adjusted to 3.0, and then NaAc-HAc buffer solution was used for dilution to obtain the solution of various concentrations (0.5–9.0 mmol l^−1^). Resin (0.1 g) was added to 100 ml of the aforementioned solution, the beaker was placed onto the constant temperature oscillator, and it was oscillated at the rate of 200 r.p.m. at a constant temperature for 12 h. The adsorption isotherm of As(V), Cr(VI), Cu(II), Pb(II), Cd(II) and Cr(III) was plotted using *Q*_e_ versus *C*_e_.

#### 2.5.3. Adsorption kinetics

Resin (0.1 g) and 100 ml of As(V), Cr(VI), Cu(II), Pb(II), Cd(II) and Cr(III) of an initial concentration of 7 mmol l^−1^ (the pH value of Cu(II), Pb(II), Cd(II) and Cr(III) was 5.0, that of As(V) was 4.0, and that of Cr(VI) was 3.0) were added to a series of 250 ml conical flasks, then the flasks were placed onto the constant temperature oscillators, constant temperature oscillation was performed at 200 r.p.m. at 25°C, 1 ml of solution was taken out every a certain period of time, the atomic absorption spectometry (AAS) was used to determine the concentration of metal ions in the solution, and the adsorption capacity of the resin was calculated. A dynamical parameter curve was plotted by adsorption capacity (*Q*) versus adsorption time (*t*).

### 2.6. Adsorption selectivity

The adsorption selectivity of the resin was studied under the condition of competition; a binary system consisting of 0.1 g of resin and 100 ml of As(V), (initial concentration 7 mmol l^−1^, pH 4.0), Cr(VI) (initial concentration 7 mmol l^−1^, pH 3.0), Cu(II), Pb(II), Cd(II) and Cr(III) (initial concentration 7 mmol l^−1^, pH 5.0) and coexisting ions (Na(I), K(I), Ca(II), Mg(II), Fe(III) and Zn(II)) (initial concentration 14 mmol l^−1^) was added into a series of 250 ml conical flasks, then the flasks were placed onto the constant temperature oscillators, and constant temperature oscillation was performed at 200 r.p.m. at 25°C. The determination method of adsorptive capacity was the same as the aforementioned determination method.

### 2.7. Regeneration

The performance of repeated use is a very important index or parameter to measure whether the adsorption materials are of application value. The adsorption materials with saturation adsorption of Cu(II), Pb(II), Cd(II) and Cr(III) were put into 0.1 mol l^−1^ HNO_3_ solution, the adsorption materials with saturation adsorption of As(V) and Cr(VI) were put into the 0.1 mol l^−1^HNO_3_ solution for stirring at room temperature for 5 h to make the metal ions adsorbed on the adsorption materials desorbed. To further determine the performance of repeated use of adsorption materials, 10 adsorption–desorption tests were carried out repeatedly for the same adsorption material.

## 3. Results and discussion

### 3.1. Synthesis of polyamine chelating resin

**Figure 1.**
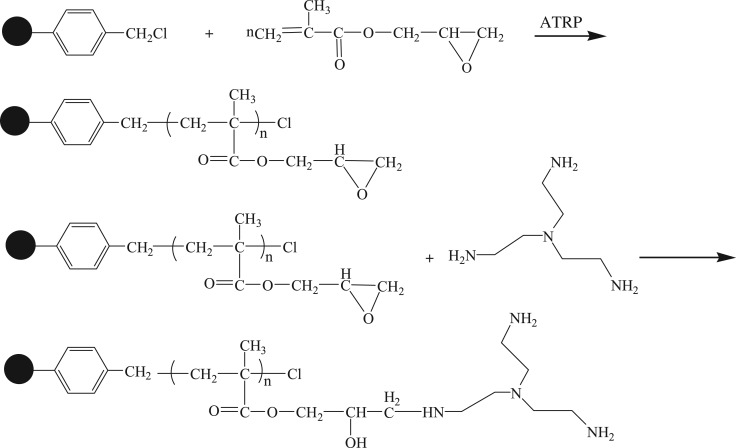
The synthetic route for the preparation of the polyamine-type chelating resin.

The two-step method was adopted for the synthesis of polyamine chelating resin, as shown in figure 1. The first step was to adopt the surface-initiated atom transfer radical polymerization to graft the GMA onto the resin surface, and introduce the epoxy group. The influences of ATRP time and different GMA consumptions on the DG of the product PS-g-PGMA are shown in the literature [18].

**Figure 2.**
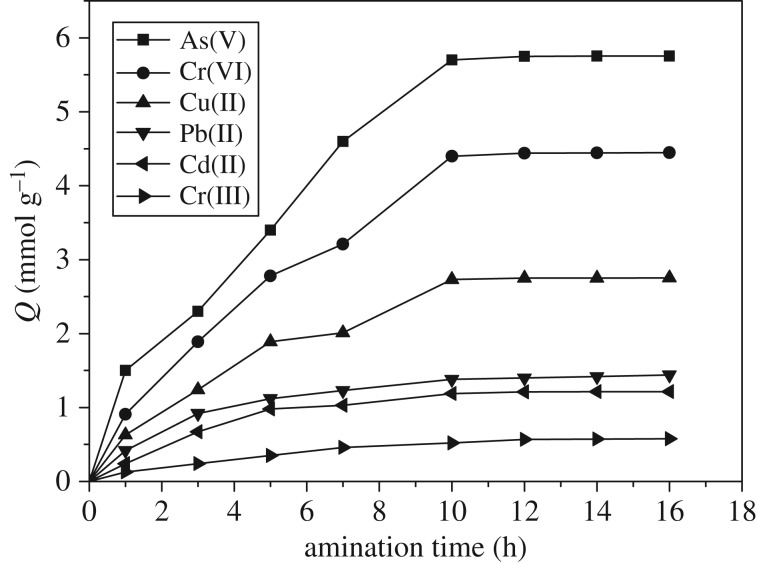
Effects of the amination reaction time on the sorption capacity.

The second step was to perform amination between the amino intris(aminoethyl)amine and the epoxy group in GMA to obtain the polyamine chelating resin. Figure 2 provides the influences of the resin obtained at different ring opening time on the adsorption capacity of As(V), Cr(VI), Cu(II), Pb(II), Cd(II) and Cr(III). Within 7 h after the initiation of the ring opening reaction, the adsorption capacity increased rapidly, and the adsorption balance was reached at approximately 10 h. As amino was mainly responsible for the adsorption of metal ions, the increase in adsorption capacity indicated that more amino groups of tris(aminoethyl)amine were introduced onto the resin surface through the reaction with the epoxy group in GMA. Therefore, the ring opening reaction duration was fixed as 12 h to prepare the polyamine chelating resin needed for the subsequent adsorption tests.

### 3.2. Resin characterization

**Figure 3.**
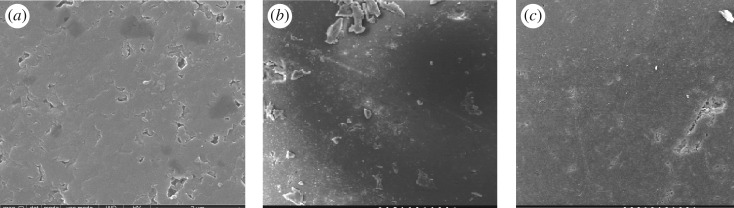
SEM micrograph of (*a*) CMPS, (*b*) PS-g-PGMA and (*c*) polyamine-type chelating resin.

SEM was used to observe the shape and appearance of the resin before and after ATRP and amination reaction. Figure 3*c* shows the topography of the polyamine chelating resin after the ring opening reaction, which is completely different from the surface of the resin after the ATRP reaction (figure 3*b*). Owing to the introduction of resin surface amino, the surface hydrophilicity of polyamine resin increased, further leading to the changes in surface topography.

**Figure 4.**
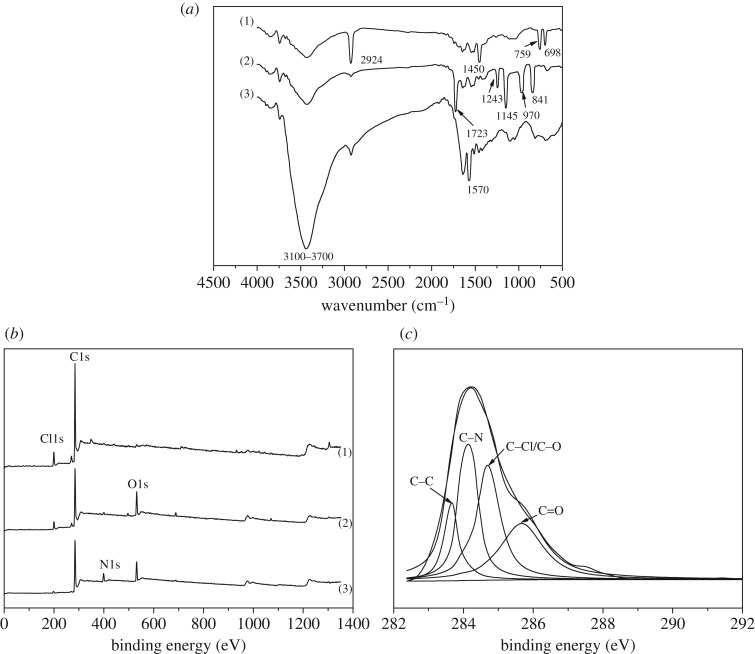
Resin characterization by FTIR A and XPS. (*a*) FTIR spectrum of (1) CMPS, (2) PS-g-PGMA and (3) polyamine-type chelating resin. (*b*) XPS wide scan of (1) CMPS, (2) PS-g-PGMA and (3) polyamine-type chelating resin. (*c*) C1 s core-level spectra of poly-amine-type chelating resin.

To verify the reactions, an analysis was made on the infrared spectrogram of the resin before and after modification, and figure 4*a* shows the infrared spectrogram of the resin before and after modification.

The characteristic absorption peaks at 2924 and1450 cm^−1^ represented the –CH_2_– stretching vibration and aromatic ring skeleton vibration, and the characteristic absorption peaks at 759 and 698 cm^−1^ represented the stretching vibration of the C–Cl in –CH_2_Cl. After the surface modification of resin, the comparison with figure 4*a*(1) indicates that significant changes occur to the characteristic absorption peak in figure 4*a*(2) and 4*a*(3). In figure 4*a*(2), the peak at 1723 cm^−1^ corresponds to the stretching vibration absorption peak with C=O in GMA, the peaks at 1243 cm^−1^ and 1145 cm^−1^ correspond to the symmetric and asymmetric vibration with C─O in GMA, and the characteristic absorption peaks of epoxy chain appear at 907 cm^−1^ and 841 cm^−1^, indicating that PGMA had been grafted onto the resin surface. In figure 4*a*(3), the characteristic absorption peaks of epoxy chain at 907 cm^−1^ and 841 cm^−1^ almost disappeared after the ring opening reaction between epoxy group and amino. After the amination reaction, broad peaks appeared at the peak within the range of 3100–3700 cm^−1^, which often corresponded to the stretching vibration of NH_2_, indicating that amino had been successfully introduced to the surface of polyamine resin. One new absorption peak appeared at 1570 cm^−1^, which corresponded to the deformation vibration of NH_2_, further verifying that amino was successfully introduced to the polyamine resin surface.

XPS was also used to analyse the surface composition and the changes in functional groups of the resin before and after ATRP and amination reactions. Figure 4*b*,*c* shows the XPS wide spectral scanogram of the resin before and after the modification, and the peak separation map of polyamine resin C 1s. In figure 4*b*(3), The N 1s peak appeared at 397.2 eV, indicating that amino was successfully introduced through the amination reaction. In the polyamine resin C 1s peak separation map, four peaks appeared at 283.7 eV, 284.9 eV, 285.4 eV and 85.9 eV as shown in figure 4*c*, which corresponded to C–C, C–N, C–Cl/C–O and C=O.

The element analysis was adopted to determine the element content of resin (table 1). After the amination reaction, the N content in resin increased rapidly, indicating that amino was introduced through the ring opening reaction.

**Table 1.** Elemental compositions of resins.

	elemental content/%		
sample	C	H	N
CMPS	56.67	8.690	0.455
PS-PGMA(ATRP10 h)	68.8	4.769	0.076
polyamine-type chelating resin	69.45	6.880	8.364

All these results indicated that GMA was successfully grafted onto the polystyrene resin through the surface-initiated atom transfer radical polymerization, and the amino group was successfully introduced into the molecular chain through the ring opening reaction between amino and epoxy groups.

### 3.3. Adsorption of heavy metal ions

#### 3.3.1. Effect of pH

**Figure 5.**
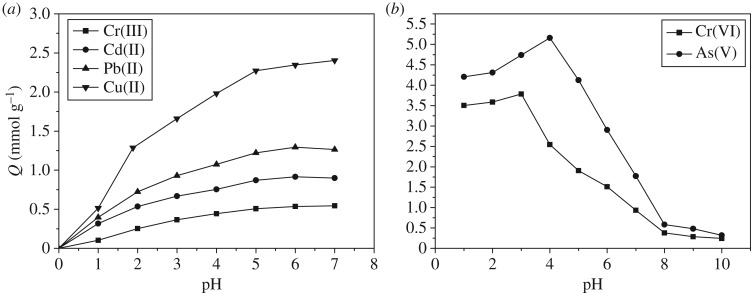
Effect of pH on the adsorption of polyamine-type chelating resin for (*a*) Cu(II), Pb(II), Cd(II) and Cr(III), and (*b*) As(V) and Cr(VI) (initial concentration: 7 mmol l^−1^; 25°C; adsorbent dose: 0.1 g).

The polyamine chelating resin obtained from the reaction of SI-ATRP and amination reaction was used for the adsorption of As(V), Cr(VI), Cu(II), Pb(II), Cd(II) and Cr(III). The pH value of the solution would affect the existence form of metal ions in the solution, the changes in the ion form of the resin surface functional groups as well as the competitive effects of the hydrogen ions and metal ions in the solution. Figure 5 shows the influences of pH value on the adsorption capacity.

To avoid the generation of hydroxide precipitates, the pH values of Cu(II), Pb(II), Cd(II) and Cr(III) solutions were controlled to be below 7.0. As shown in figure 5*a*, the adsorption capacity of resin on Cu(II), Pb(II), Cd(II) and Cr(III) increased with the increase in pH value. In the case of a pH value of less than 2.0, resin almost had no adsorption on Cu(II), Pb(II), Cd(II) and Cr(III), because in the case of a relatively low pH value, the amino group would be protonated and the electrostatic repulsion reaction made the positive ions unable to approach the adsorption sites. With the increase in pH value, some amino groups in the resin adsorbed Cu(II), Pb(II), Cd(II) and Cr(III) through chelation. In the case of a low pH value, the adsorption capacity was low, so acid could be used to regenerate the resin that had adsorbed Cu(II), Pb(II), Cd(II) and Cr(III).

Figure 5*b* shows the influences of pH value on the adsorption of polyamine chelating resin on negative ions As(V) and Cr(VI). In the case of a pH value of more than 2.0, the adsorption capacity of the resin on Cr(VI) decreased; in the case of a pH of 3.0, the adsorption capacity was 3.78 mmol g^−1^; in the case of a pH of 10.0, the adsorption capacity was reduced to 0.24 mmol g^−1^. The adsorption capacity on As(V) started to increase; when the pH value was greater than 4.0, it decreased gradually; when the pH value was 10.0, there was almost no adsorption. When the pH value was 2.0, most As(V) was present in the form of H_3_AsO_4_ [19]; therefore, it could not be adsorbed by the protonated amino groups through the electrostatic interactions; when the pH value was less than 3, the adsorption capacity was relatively low. When the pH value was within the range of 4.0–10.0, As(V) was present in the form of ${\text{H}}_{2}{\text{AsO}}_{4}^{-}$, ${\text{HAsO}}_{4}^{2}$ and ${\text{AsO}}_{4}^{3-}$ [19]; during the adsorption of the protonated amino resin on the negative ions As(V), electrostatic attraction played an important role. With the increase in the pH value of the solution, the number of protonated amino groups decreased, leading to a decrease in the adsorption capacity of As(V). Likewise, when the pH value was greater than 3.0, Cr(VI) was present in the form of negative ions (${\text{HCrO}}_{4}^{-}$, ${\text{CrO}}_{4}^{2-}$ and $\text{C}{\text{r}}_{2}{\text{O}}_{7}^{2-}$) [20], and the adsorption process mainly depended on the electrostatic interactions. Actually, the different adsorption behaviours of amino resin on negative ions and positive ions indicated different adsorption mechanisms during the adsorption process, which will be discussed in the following section.

#### 3.3.2. Adsorption kinetics

**Figure 6.**
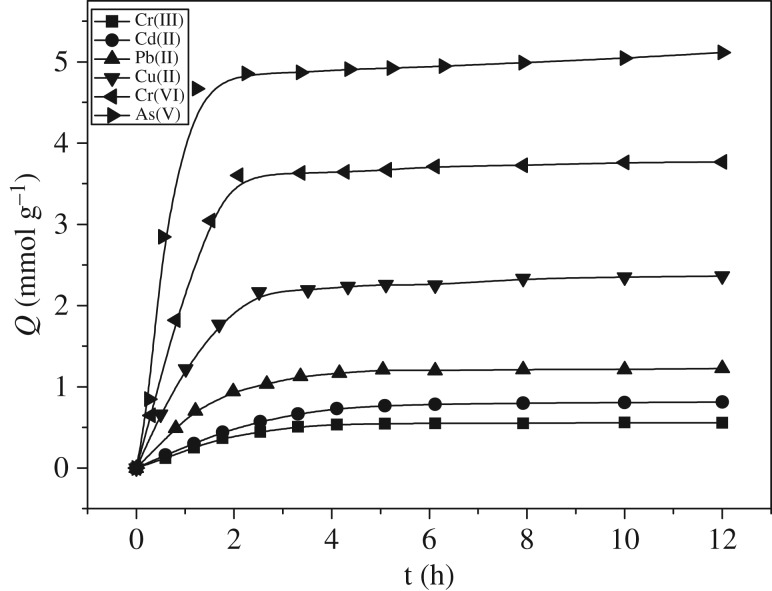
Adsorption kinetics of polyamine-type chelating resin for As(V), Cr(VI), Cu(II), Pb(II), Cd(II) and Cr(III) (initial concentration: 7 mmol l^−1^; 25°C; As(V) pH 4.0; Cr(VI) pH 3.0; Cu(II), Pb(II), Cd(II) and Cr(III) pH 5.0; adsorbent dose: 0.1 g).

Figure 6 shows the kinetic curve of the adsorption of polyamine chelating resin on As(V), Cr(VI), Cu(II), Pb(II), Cd(II) and Cr(III). As shown in the figure, amino resin had the fastest adsorption of As(V), the time needed to reach the adsorption balance was as follows: 1 h for As(V), 2 h for Cr(VI), 2.5 h for Cu(II), 3 h for Pb(II), 5 h for Cd(II) and 6 h for Cr(III). In spite of the same initial concentration of various metal ions (7 mmol l^−1^), they had different adsorption capacities when the adsorption balance was reached. The adsorption capacity of As(V) was as high as 5.16 mmol g^−1^, followed by Cr(VI), Cu(II), Pb(II), Cd(II) and Cr(III). Within the first 60 min, the adsorption capacity of various kinds of metal ions increased significantly, and gradually the adsorption balance was reached.

The data in figure 8 were put into the kinetic equation of quasi-secondary reaction

3.1 $$\frac{t}{Q_{t}}=\frac{1}{kQ_{e}^{2}}+\frac{1}{Q_{e}}t,$$

where *t* represents the adsorption time (hours), *k* is the adsorption rate constant (g mmol^−1^ h^−1^), and *Q_t_* and *Q*_e_ represent the time *t* and the adsorptive capacity when the equilibrium is reached (mmol g^−1^).

Formula (3.1) was used for the fitting of the adsorption kinetic data, and the finding was that the linear correlation coefficient *R*^2^ was greater than 0.998, indicating that the adsorption of resin on As(V), Cr(VI), Cu(II), Pb(II), Cd(II) and Cr(III) conformed to the quasi-secondary kinetic model, and that the adsorption process was the electrostatic interaction between the adsorbent and the adsorbate or the chemical adsorption induced by coordination.

Through the intercept and the slope of the straight line, the reaction rate constants of As(V), Cr(VI), Cu(II), Pb(II), Cd(II) and Cr(III) were obtained (1.006, 1.023, 2.238, 0.776, 1.455 and 0.834 g mmol^−1^ h^−1^, respectively).

#### 3.3.3. Adsorption isotherm

**Figure 7.**
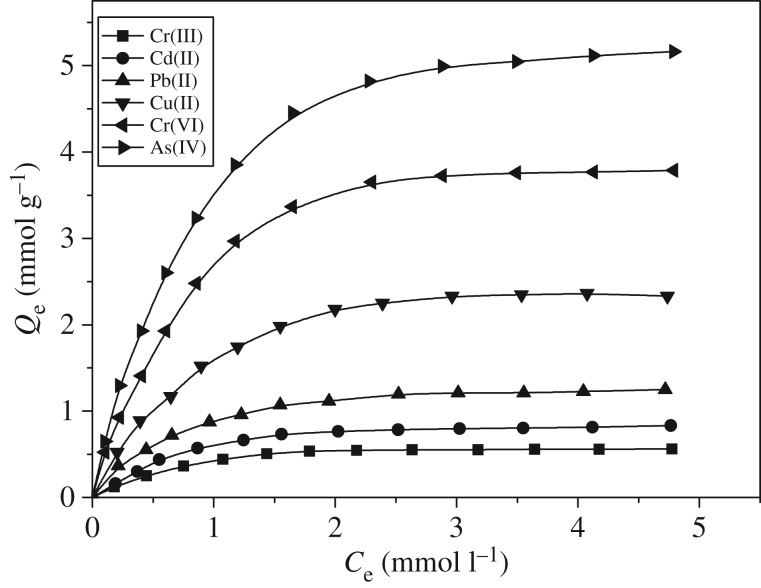
Adsorption isotherms of polyamine-type chelating resin for As(V), Cr(VI), Cu(II), Pb(II), Cd(II) and Cr(III) at 25°C (As(V) pH 4.0; Cr(VI) pH 3.0; Cu(II), Pb(II), Cd(II) and Cr(III) pH 5.0; contact time: 12 h; adsorbent dose: 0.1 g).

Adsorption isotherm, characterized with some constants, can be used to explain the surface nature and affinity of the resin, and can also be used to compare the adsorption capacity of metal ions. Figure 7 shows the isotherm of the adsorption of 25°C polyamine resin on As(V), Cr(VI), Cu(II), Pb(II), Cd(II) and Cr(III).

The adsorption experimental data were analysed separately using the Langmuir model (3.2) and Freundlich (3.3) model.

3.2 $$\frac{C_{e}}{Q_{e}}=\frac{1}{Q_{0}K_{C}}+\left(\frac{1}{Q_{0}}\right)C_{e}$$

and

3.3 $$\ln Q_{e}=\ln K_{F}+\frac{1}{n}\ln C_{e},$$

where *Q*_e_ is the adsorption capacity, mmol g^−1^; *C*_e_ is the equilibrium concentration of metal ions, mmol l^−1^; *Q*_0_ is the saturated adsorption capacity, mmol g^−1^; *K*_C_ is the Langmuir constant, l mmol^−1^; *K*_F_ is an empirical parameter, mmol^1–1/*n*^ l^−1/*n*^ g^−1^; *n* is the Freundlich constant; and *K*_F_ is the binding energy constant reflecting the affinity of the adsorbents to metal ions. As shown in table 2, the linear correlation coefficient obtained using the Langmuir fitting was superior to the results obtained using the Freundlich, indicating that the adsorption of As(V), Cr(VI), Cu(II), Pb(II), Cd(II) and Cr(III) onto the polyamine resin belonged to the Langmuir monomolecular layer absorption model, which is an inevitable result induced by the chemical adsorption process.

**Table 2.** Langmuir and Freundlich constants for As(V), Cr(VI), Cu(II), Pb(II), Cd(II) and Cr(III) adsorption on polyamine-type chelating resin at 25°C.

	Langmuir parameters			Freundlich parameters		
metal ions	*Q*_0_ (mmol g^−1^)	*K*_c_	$R_{L}^{2}$	*K*_F_	1/*n*	$R_{F}^{2}$
As(V)	5.75	77.67	0.99412	3.136	0.6196	0.9456
Cr(VI)	4.44	64.33	0.98714	2.358	0.6213	0.950
Cu(II)	2.75	63.22	0.98377	0.368	0.52954	0.91015
Pb(II)	1.40	75.44	0.99469	0.541	0.56431	0.94345
Cd(II)	1.21	58.78	0.98159	0.868	0.62172	0.95957
Cr(III)	0.57	61	0.99761	1.47	0.5424	0.9755

The maximal adsorption capacity of polyamine resin on As(V), Cr(VI), Cu(II), Pb(II), Cd(II) and Cr(III) was 5.75, 4.44, 2.75, 1.40, 1.21 and 0.57 mmol g^−1^, respectively; when compared with other resins (table 3), apparently our synthesized polyamine chelating resin had a higher adsorptive capacity on As(V), Cr(VI), Cu(II), Pb(II), Cd(II) and Cr(III) than the adsorbent synthesized by traditional methods, and also than the resin prepared by the combination of ATRP and dual-functional chelating agent. This indicates that the combination of ATRP and hyperbranching concept is an effective method for the preparation of high capacity chelating resin.

**Table 3.** Comparison of As(V), Cr(VI), Cu(II), Pb(II), Cd(II) and Cr(III) adsorption on polyamine-type chelating resin with other adsorbents.

	adsorption capacities (mmol g^−1^)						
adsorbents	As(V)	Cr(VI)	Cu(II)	Pb(II)	Cd(II)	Cr(III)	ref.
aminated resin prepared via surface-initiated atom transfer radical polymerization (SI-ATRP) and subsequent amination reaction	2.2	3.0	2.6	0.97	—	—	[10]
functionalized SBA-15 mesoporous silica by melamine-based dendrimer amines	—	—	0.59	0.20	0.53	—	[21]
Na-montmorillonite	—	—	0.11	0.12	—	0.20	[22]
peat	—	—	0.13	0.19	—	0.25	[23]
growing and non-growing cells of a bacterial consortium	—	1.75	2.79	1.15	—	—	[24]
silica-supported bis(diazoimine) ligands	—	—	0.003	0.001	0.0018	0.0026	[25]
activated carbon that was modified with rhodamine 6G	—	—	0.75	0.97	0.20	0.73	[26]
*N*,*N*-dimethylaminoethylmethacrylate (DMAEMA) grafted polyethylene/polypropylene(PE/PP) non-woven fibres (DMAEMA-g-PE/PP)	1.11	—	—	—	—	—	[27]
amino starch preparation	—	0.24	0.46	—	—	—	[28]
biomass-based hydrogel	—	—	1.18	—	—	0.80	[29]
a two-dimensional porous Fe_2_O_3_/graphitic-C_3_N_4_/graphene ternary nanocomposite	—	2.86	—	—	—	—	[30]
polyamine-type chelating resin	5.75	4.44	2.75	1.40	1.21	0.57	this work

#### 3.3.4. Adsorption thermodynamics

**Figure 8.**
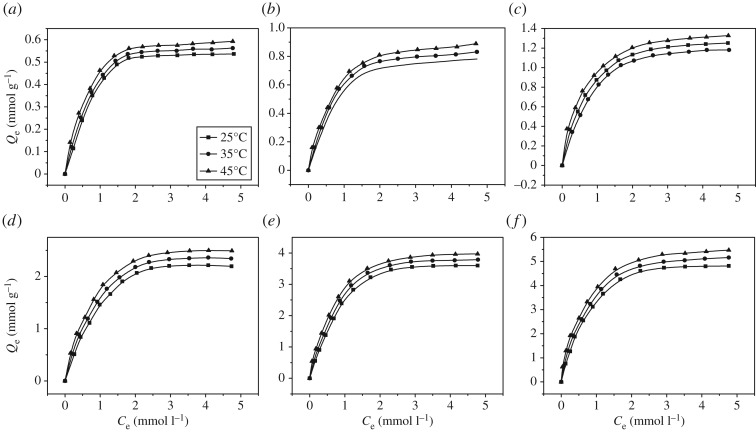
Adsorption isotherms of polyamine-type chelating resin for (*a*) Cr(III), (*b*) Cd(II), (*c*) Pb(II), (*d*) Cu(II), (*e*) Cr(VI) and (*f*) As(V) at different temperature (As(V) pH 4.0; Cr(VI) pH 3.0; Cu(II), Pb(II), Cd(II) and Cr(III) pH 5.0; contact time: 12 h ; adsorbent dose: 0.1 g).

Figure 8 shows the influences of temperature on the adsorption of polyamine chelating resin on As(V), Cr(VI), Cu(II), Pb(II), Cd(II) and Cr(III). Gibbs free energy (Δ*G*), enthalpy change (Δ*H*) and enthalpy change (Δ*S*) were obtained through the calculation using

3.4 $$\begin{array}{r} \Delta G=-RT\ln K_{C}, \end{array}$$

3.5 $$\begin{array}{r} \Delta G=\Delta H-T\Delta S \end{array}$$

3.6 $$\begin{array}{r} \;\text{and}\;\text{ln}\;K_{C}=\frac{-\Delta H}{RT}+\frac{\Delta S}{R}, \end{array}$$

and the results are shown in table 4.

**Table 4.** Thermodynamic parameters estimated for adsorption of six metal ions on polyamine-type chelating resin.

			▵*G* (kJ mol^−1^)		
metal ions	▵*H* (kJ mol^−1^)	▵*S* (J mol^−1^ K^−1^)	25°C	35°C	45°C
As(V)	12.02	76.5	−10.78	−11.47	−12.46
Cr(VI)	14.56	83.5	−10.32	−11.11	−12.08
Cu(II)	11.57	73.3	−10.27	−10.99	−11.76
Pb(II)	15.60	88.6	−10.71	−11.58	−12.52
Cd(II)	15.54	86	−10.09	−10.94	−11.83
Cr(III)	5.70	53.3	−10.18	−10.73	−11.21

With the increase in temperature, the adsorption capacity of all metal ions increased somewhat; the probable reason was that the increase in temperature made the binding site become active and the activity of metal ions increases so that the ions were more easily adsorbed on the resin surface. A negative value of Δ*G* and a positive value of Δ*H* mean that the adsorption process of polyamine chelating resin on As(V), Cr(VI), Cu(II), Pb(II), Cd(II) and Cr(III) was a spontaneous heat absorption process, and a positive value of Δ*S* meant the adsorption process was an enthalpy-driven spontaneous process.

#### 3.3.5. Adsorption mechanism

**Figure 9.**
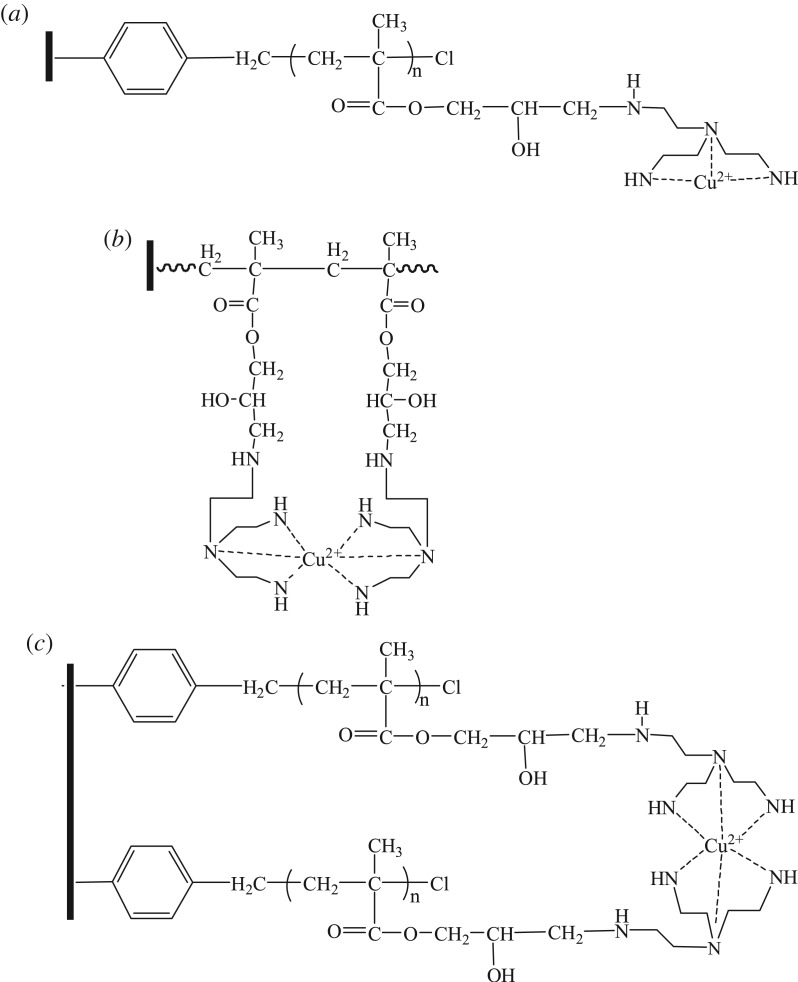
Adsorption mechanism of polyamine-type chelating resin for Cu(II).

Polyamine chelating resin adsorbed Cu(II), Pb(II), Cd(II) and Cr(III) through chelation. Owing to the adoption of ATRP technology, the functional groups introduced onto the resin surface had a high density, and one metal ion might be surrounded by three amino groups in one polymer chain (figure 9*a*), or multiple amino groups in six neighbouring amino groups (figure 9*b*) and two neighbouring polymer chains (figure 9*c*). Therefore, one metal ion could bind with three or six amino groups through coordination (with Cu^2+^ as the example).

**Figure 10.**
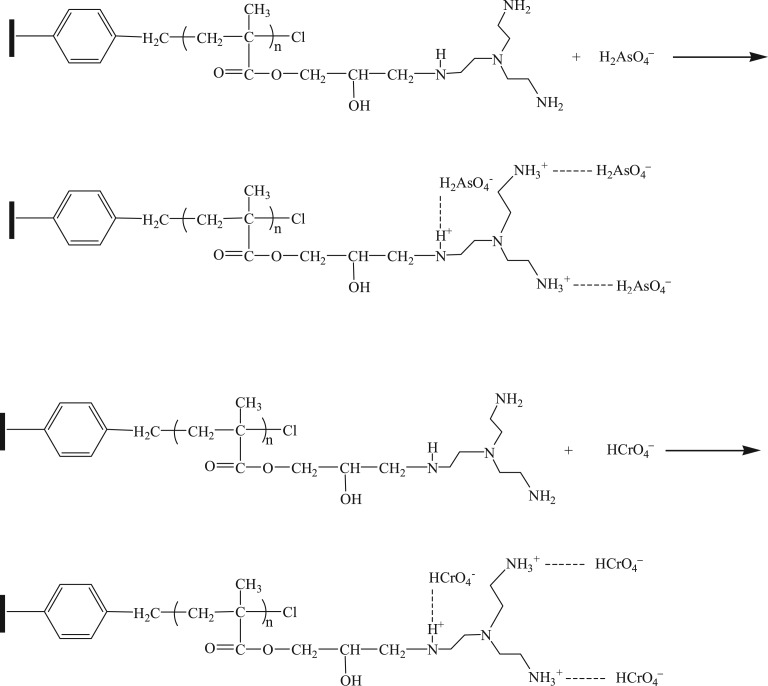
Adsorption mechanism of polyamine-type chelating resin for As(V) and Cr(VI).

Protonated amino groups were responsible for the adsorption of As(V) and Cr(VI); in the case of a pH of 4.0, As(V) (H_2_AsO_4_^−^) was adsorbed through the electrostatic attraction, as shown in figure 10 (with ${\text{H}}_{2}{\text{AsO}}_{4}^{-}$ as the example).

In the meantime, in the case of a pH value of 3.0, ${\text{HCrO}}_{4}^{-}$ was the primary existence form, and protonated amino groups adsorbed Cr(VI) ${\text{( HCrO}}_{4}^{-})$ through electrostatic interaction, as shown in figure 10 (with ${\text{HCrO}}_{4}^{-}$ as the example).

### 3.4. Adsorption selectivity

Na(I), K(I), Ca(II), Mg(II), Fe(III) and Zn(II) are essential elements for organisms, and the adsorption of chelating resin on harmful metal ions will affect the bioavailability of these ions. In the meantime, the presence of such essential elements in aqueous solution is destined to affect the adsorption performance of chelating resin on the harmful metal ions. Therefore, we studied the influences of the coexistence of these ions on the adsorption on As(V), Cr(VI), Cu(II), Pb(II), Cd(II) and Cr(III). The following solutions were added to 0.1 g of polyamine chelating resin for adsorption: 100.0 ml of the solution containing Cu(II), Pb(II), Cd(II) and Cr(III) (the concentration was 7.0 mmol l^−1^) and each kind of coexisting ions (the concentration was 14.0 mmol l^−1^) with a pH value of 5.0, 100.0 ml of the solution containing As(V) (the concentration was 7.0 mmol l^−1^) and each kind of coexisting ions (the concentration was 14.0 mmol l^−1^) with a pH value of 4.0, and 100.0 ml of the solution containing Cr(VI) (the concentration was 7.0 mmol l^−1^) and each kind of coexisting ions (the concentration was 14.0 mmol l^−1^) with a pH value of 3.0. As shown in table 5, the influences of the corresponding pH values of Na(I), K(I), Ca(II), Mg(II), Fe(III) and Zn(II) upon the adsorption on As(V) and Cr(VI) can be neglected; the main reason is that, in the case of a pH value of 3.0 and 4.0, the amino groups are protonated and positively charged, so they cannot adsorb Na(I), K(I), Ca(II), Mg(II), Fe(III) and Zn(II) through electrostatic interaction. The influences of Na(I) and K(I) upon the adsorption on Cu(II), Pb(II), Cd(II) and Cr(III) can be neglected, and Ca(II), Mg(II), Fe(III) and Zn(II) can somehow affect the adsorption on Cu(II), Pb(II), Cd(II) and Cr(III). This phenomenon can be used to explain the theory of HSAB. As shown in the obtained results, amino has the soft base nature; correspondingly, when compared with Na(I), K(I), Ca(II) and Mg(II) (hard acid), Fe(III) and Zn(II) are more apt to bind with soft base, which further affect the adsorption of resin on Cu(II), Pb(II), Cd(II) and Cr(III). However, the adsorption capacity of polyamine chelating resin on Cu(II), Pb(II), Cd(II) and Cr(III) far exceeds that of Fe(III) and Zn(II). These results indicate that polyamine chelating resin can selectively adsorb As(V), Cr(VI), Cu(II), Pb(II), Cd(II) and Cr(III) in the waste water.

**Table 5.** Effect of coexisting metal ions on the adsorption capacity (As(V) pH 4.0; Cr(VI) pH 3.0; Cu(II), Pb(II), Cd(II) and Cr(III) pH 5.0; contact time: 12 h; adsorbent dose: 0.1 g).

	selective coefficients					
coexisting ions	As(V)	Cr(VI)	Cu(II)	Pb(II)	Cd(III)	Cr(III)
K^+^	∞	∞	∞	∞	∞	∞
Na^+^	∞	∞	∞	∞	∞	∞
Ca^2+^	∞	∞	88.6	65.4	52.1	44.1
Mg^2+^	∞	∞	75.3	45.2	32.1	29.8
Fe^3+^	∞	∞	32.2	25.6	20.1	18.6
Zn^2+^	∞	∞	21.3	16.7	13.2	11.4

### 3.5. Reusability and stability

The resin which had adsorbed Cu(II), Pb(II), Cd(II) and Cr(III) was placed into the 0.1 mol l^−1^HNO_3_ solution for desorption, and it turned out that the desorption rate was over 97%. The resin which had adsorbed As(V) and Cr(VI) was placed into the 0.1 mol l^−1^HNO_3_ solution for desorption, and it turned out that the desorption rate is over 98%. To investigate the regeneration performance of resin, the metal ions were adsorbed and desorbed by using the same method to repeat the process ten times. As shown in table 6, after ten adsorption–desorption cycles, the adsorption capacity on As(V) decreased only by 2.78%, that on Cr(VI) decreased by 3.83%, that on Cu(II) decreased by 6.11%, that on Pb(II) decreased by 9.29%, that on Cd(II) decreased by 3.09%, and that on Cr(III) decreased by 8.77%, indicating no significant change in the adsorption capacity after ten adsorption–adsorption cycles.

**Table 6:** Adsorption and desorption behaviours of As(V), Cr(VI), Cu(II), Pb(II), Cd(II) and Cr(III) on polyamine-type chelating resin.

	adsorption capacities (mmol g^−1^)					
cycle	As(V)	Cr(VI)	Cu(II)	Pb(II)	Cd(II)	Cr(III)
1	5.75	4.44	2.75	1.40	1.21	0.57
2	5.72	4.41	2.71	1.38	1.20	0.55
3	5.69	4.39	2.68	1.36	1.18	0.56
4	5.70	4.40	2.69	1.39	1.19	0.54
5	5.74	4.38	2.65	1.35	1.16	0.57
6	5.71	4.36	2.62	1.37	1.17	0.53
7	5.68	4.37	2.64	1.33	1.15	0.52
8	5.66	4.33	2.61	1.30	1.13	0.53
9	5.62	4.31	2.58	1.28	1.10	0.53
10	5.59	4.27	2.54	1.27	1.09	0.52

## 4. Conclusion

By means of SI-ATRP and the polyamine reactions with hyperbranched structures, we have successfully prepared a new kind of polyamine chelating resin with hyperbranched structures for the adsorption of heavy metal ions in solution. The finding is that the adsorptive capacity of resin on the studied heavy metal ions is higher than that of the resin synthesized by traditional technology and also higher than that of the chelating resin modified by ATRP technology and bifunctional chelator, indicating that the combination of ATRP and hyperbranched polymer concept is an effective method to prepare high capacity chelating resin.

This resin has a selective effect on As(V) and Cr(VI) in the case of a relatively low pH, and it can be used for the selective adsorption of As(V) and Cr(VI) in the heavy metal waste water; the resin has a relative good adsorption performance on Cu(II), Pb(II), Cd(II) and Cr(III) in the case of a relatively high pH. Through the control of the solution pH, resin has a relative adsorption performance on negative ions and positive ions, and its application is broad in the handling of heavy metal waste water.

## Data accessibility

This article has no additional data.
